# Supplementary material for: Prevalence of blindness and its determinants in Bangladeshi adult population: results from a national cross-sectional survey
Source: BMJ Open. 2022 Apr 1;12(4):e052247. doi: 10.1136/bmjopen-2021-052247 (PMC8977819; doi:10.1136/bmjopen-2021-052247)
Supplement: Author's manuscript [file bmjopen-2021-052247.draft_revisions.pdf]

BMJ Open

BMJ Open is committed to open peer review. As part of this commitment we make the peer review history of every article we publish publicly available.

When an article is published we post the peer reviewers' comments and the authors' responses online. We also post the versions of the paper that were used during peer review. These are the versions that the peer review comments apply to.

The versions of the paper that follow are the versions that were submitted during the peer review process. They are not the versions of record or the final published versions. They should not be cited or distributed as the published version of this manuscript.

BMJ Open is an open access journal and the full, final, typeset and author-corrected version of record of the manuscript is available on our site with no access controls, subscription charges or pay-per-view fees (http://bmjopen.bmj.com).

If you have any questions on BMJ Open's open peer review process please email info.bmjopen@bmj.com

BMJ Open

Prevalence of blindness and its determinants in Bangladeshi adult population: Results from a national cross-sectional survey

Journal:	*BMJ Open*
Manuscript ID	bmjopen-2021-052247
Article Type:	Original research
Date Submitted by the Author:	12-Apr-2021

Complete List of Authors:	Ara Shakoor, Shawkat; National Institute of Opthalmology<br>Rahman, Mustafizur ; Dhaka Medical College and Hospital<br>Hossain, A.H.M. Enayet; National Institute of Opthalmology<br>Moniruzzaman, Mohammad; Shiga University of Medical Science, Public Health<br>Bhuiyan, Mahfuzur ; National Heart Foundation of Bangladesh<br>Hakim, Ferdous; World Health Organization Regional Office for South-East Asia, Country Office for Bangladesh<br>Zaman, MM; World Health Organization Regional Office for South-East Asia, Country Office for Bangladesh

Keywords:	Cataract and refractive surgery < OPHTHALMOLOGY, Glaucoma < OPHTHALMOLOGY, EPIDEMIOLOGY, OPHTHALMOLOGY, Corneal and external diseases < OPHTHALMOLOGY

SCHOLARONE™
Manuscripts

I, the Submitting Author has the right to grant and does grant on behalf of all authors of the Work (as defined in the below author licence), an exclusive licence and/or a non-exclusive licence for contributions from authors who are: i) UK Crown employees; ii) where BMJ has agreed a CC-BY licence shall apply, and/or iii) in accordance with the terms applicable for US Federal Government officers or employees acting as part of their official duties; on a worldwide, perpetual, irrevocable, royalty-free basis to BMJ Publishing Group Ltd ("BMJ") its licensees and where the relevant Journal is co-owned by BMJ to the co-owners of the Journal, to publish the Work in this journal and any other BMJ products and to exploit all rights, as set out in our licence.

The Submitting Author accepts and understands that any supply made under these terms is made by BMJ to the Submitting Author unless you are acting as an employee on behalf of your employer or a postgraduate student of an affiliated institution which is paying any applicable article publishing charge ("APC") for Open Access articles. Where the Submitting Author wishes to make the Work available on an Open Access basis (and intends to pay the relevant APC), the terms of reuse of such Open Access shall be governed by a Creative Commons licence – details of these licences and which Creative Commons licence will apply to this Work are set out in our licence referred to above.

Other than as permitted in any relevant BMJ Author's Self Archiving Policies, I confirm this Work has not been accepted for publication elsewhere, is not being considered for publication elsewhere and does not duplicate material already published. I confirm all authors consent to publication of this Work and authorise the granting of this licence.

For peer review only

Prevalence of blindness and its determinants in Bangladeshi adult population: Results from a national cross-sectional survey

Shawkat Ara Shakoor[1], MScCEH, MS; ORCID: https://orcid.org/0000-0001-6299-7529

Mustafizur Rahman[2], MBBS; MS; ORCID: https://orcid.org/0000-0002-7199-1070

A H M Enayet Hussain[3], FCPS, FRCS; ORCID: https://orcid.org/0000-0003-2715-5574

Mohammad Moniruzzaman[4], MPhil, PhD; ORCID: https://orcid.org/0000-0003-2144-7111;

Mahfuzur Rahman Bhuiyan[5], MBBS, MPH; ORCID: https://orcid.org/0000-0001-6962-7264;

Ferdous Hakim[6], MBBS, MPH; ORCID: https://orcid.org/0000-0003-2376-3978;

M Mostafa Zaman[7], MBBS, PhD; ORCID: https://orcid.org/0000-0002-1736-1342

Affiliations:

[1]National Institute of Ophthalmology, Dhaka, Bangladesh; Email: millyshakoor65@gmail.com

[2]Dhaka Medical College, Dhaka, Bangladesh. Email: mdmustafizur.ophth@gmail.com

[3]Directorate General of Health Services, Dhaka, Bangladesh; Email: paedeye@yahoo.com

[4]Shiga University of Medical Science, Shiga, Japan; Email: mmzbd82@gmail.com

[5]National Heart Foundation of Bangladesh, Dhaka, Bangladesh; Email: mahfuzdoc@yahoo.com

[6, 7]World Health Organization, Dhaka, Bangladesh; Email: hakimf@who.int; zamanm@who.int

Running head: Blindness in Bangladeshi adults

Word count: Main text: 2100, Abstract: 286

Tables: 3

Figures: 2

Address for correspondence:

Professor Dr M Mostafa Zaman, Adviser, Research and Publication, World Health Organization, 10 Gulshan Avenue, Road Number 5, Gulshan 1, Dhaka 1212, Bangladesh. Phone: +88028831415, Mobile: +8801714165205, Fax: +88028831423, Email: zamanm@who.int

For peer review only - http://bmjopen.bmj.com/site/about/guidelines.xhtml

For peer review only

ABSTRACT

Objective:

The objective of this study was to determine the prevalence of blindness and its determinants in Bangladeshi adult population.

Study design:

A cross-sectional population-based survey conducted at household level with national representation. Samples were drawn from the national census frame using a multistage stratified cluster sampling method.

Setting and participants:

The survey was done in urban and rural areas in 2013 using a probability proportionate to size sampling approach to locate participants from the primary sampling units. One man or one woman aged ≥40 years was randomly selected from their households to recruit 7,200. In addition to socio-demographic data, information on medication for hypertension and diabetes was obtained. Blood pressure and capillary blood glucose were measured. Eyelids, cornea, lens, and retina were examined.

Primary outcome measures:

The following definition was used to categorize subjects having: (a) blindness: visual acuity <3/60, (b) low vision: ≥3/60, and (c) normal vision: ≥6/12.

Results:

We could recruit 6,391 people (response rate 88.8%) among whom, 2955 were men and 3436 were women. Among them, 1922 were from urban and 4469 from rural areas. Overall the mean (standard deviation) age was 54.3 (11.2) years. The age-standardized prevalence, after best correction, of blindness and low vision was 1.0% (95% confidence interval, 0.5–1.4) and 12.1% (10.5–13.8) respectively. Multivariable logistic regression indicated that cataract, age-related macular degeneration, and diabetic retinopathy were significantly associated with low vision and blindness after adjustment for age and sex. Population attributable risk of cataract for low vision and blindness was 79.6%.

Conclusion:

Low vision and blindness are common problems in those aged 40 years or older. Extensive screening, and eye care services are necessary for wider coverage engaging all tiers of the health care system especially focusing on cataract.

Key words: Bangladesh, Adults, Population, Low vision, Blindness

For peer review only - http://bmjopen.bmj.com/site/about/guidelines.xhtml

Article Summary

Strengths and limitations of this study

- This nationally representative population-based survey indicates that more than 1 in 10 Bangleashi adults aged ≥40 years have low vision or blindness; cataract being the single most attributing factor.
- The study followed rigorous survey methods, including a multistage, geographically clustered, and probability proportional to size sampling approach to recruit particiapnts randomly.
- The absence of colour photos of fundus examinations might have led to bias estimate of age-related macular degeneration and diabetic retinopathy.

For peer review only

For peer review only - http://bmjopen.bmj.com/site/about/guidelines.xhtml

BACKGROUND

The impact of visual loss on an individual's personal, economic, and social life is profound. When the burden of blindness in communities is high, the consequences become a significant public health issue[1]. According to the World Health Organization (WHO), 285 million people globally live with visual impairment. Of them, 246 million have low vision, 39 million are blind, and two-thirds of this population are aged over 50 years[2]. Because of the rapid population ageing, low vision and blindness have become a global public health threat, particularly in developing countries.

Nearly 90% of the world's visually impaired people live in developing countries. The South-East Asia Region, including Bangladesh, is estimated to inhabit 90.5 million visually impaired and 12 million blind adults[3]. Globally the top four causes of visual impairment are uncorrected refractive errors, cataract, age-related macular degeneration (AMD), and glaucoma. Therefore, 80% of all visual impairments are avoidable[3].

In Bangladesh, a previous national survey—done in 2000—reported an age-standardized prevalence of blindness and low vision of 1.53% and 0.56%, respectively, among adults aged 30 years or older[4 5]. Since then, Bangladesh has passed through a remarkable demographic transition. Recent data on blindness and low vision in Bangladesh are unknown. Bangladesh has been implementing its National Eye Care[6] for preventing avoidable blindness and low vision, but mostly through tertiary level hospitals. A recent estimate was, therefore, required to inform the eye care plan and other relevant programmes. We conducted this national survey to determine the prevalence of blindness and impaired vision, and related factors in Bangladeshi adults.

METHODS

Study design, population, and setting

We conducted a nationwide population-based cross-sectional survey among Bangladeshi adults (men and women) aged 40 years or older in September—December 2013. We calculated our sample size based on a prevalence of blindness (1.53%), with a margin of error (0.00765) and a design effect of 1.5 (1483). Then we adjusted for four groups (men, women, urban and rural) and a response rate of 82.5% (7193), leaving the final sample size to 7200. The details of the sampling procedure have been described previously[7]. Briefly, we adopted a multistage, geographically clustered, probability-based sampling approach to obtain a nationally representative sample. We invited a total of 7,200 randomly selected

For peer review only - http://bmjopen.bmj.com/site/about/guidelines.xhtml

adults from 72 (urban, 25; rural, 47) primary sampling units (used in the national census) to participate from all seven divisions of Bangladesh. In each selected primary sampling unit, we identified 100 consecutive households with a random start. Then we randomly selected one participant from a list of eligible household members using the Kish table[8]. The flowchart of subject selection is given in **Figure 1.**

Patient and Public involvement

Patient and public were not involved in this study.

Data collection

Trained enumerators collected demographic, socio-economic, and medical history data using an interviewer-administered standardized questionnaire at the household level. Thereafter, they invited participants to have a physical and ophthalmic examination in a nearby health centre (or make-shift examination centre established conveniently by the research team). A team of trained ophthalmic nurses, ophthalmologists, and laboratory technologists performed these examinations. Nurses measured participants' height, weight, seated blood pressure, and (random) capillary blood glucose using a glucometer (Accu-chek Advantage, Roche Diagnostics Division, Switzerland). We used a modified WHO/PBL questionnaire Version III[9] as our instruments[10].

Ethics approval

We obtained ethical approval for this study from the Institutional Review Board of the National Institute of Ophthalmology, Dhaka, Bangladesh (Memo No. NIO/670 of 4 April 2013). All participants gave written consent through signature, if not possible, through thumbprint.

Vision and ophthalmic examinations

We used WHO International Classification of Diseases 10 categories of visual impairment for the study[11, 12]. Blindness was defined as corrected visual acuity of less than 3/60 in the better eye. Low vision was defined as corrected visual acuity of less than 6/60 but equal to or more than 3/60 in the better eye. People having visual acuity of 6/12 or more were considered to have normal vision.

Eye lids, cornea, lens (including its absence or displacement) and retina were examined. Age-related macular degeneration (AMD) was defined as the presence of any one of the

For peer review only - http://bmjopen.bmj.com/site/about/guidelines.xhtml

following: soft drusen or reticular drusen, hyper- or hypopigmentation of the retinal pigment epithelium. Diabetic retinopathy included non-proliferative, proliferative, and maculopathy subtypes. These were not mutually exclusive, as the latter two types, for example, may co-exist.

Ophthalmic nurses examined blood pressure, capillary blood glucose and took medical history of diseases such as hypertension and diabetes. Hypertension was defined as blood pressure ≥140/90 mm Hg or use of antihypertensive medicines, and diabetes was defined as casual capillary blood glucose ≥11.1 mmol/dL or use of antidiabetic medicines. Distance visual acuity was measured with Snellen 'E' chart and a hand-held tally counter, if necessary, at three meters by ophthalmic nurses. Based on presenting visual acuity, participants were assigned either a red card (acuity worse than 6/12 in either eye) or a green card (equal or better than 6/12 in both eyes tested separately).

Intra-ocular pressure was measured using Schiotz tonometer after application of Tetracaine hydrochloride (1%). A relative afferent pupil defect in those patients with a best-corrected visual acuity of <6/12 in either eye was tested. The ophthalmologist assessed the fundus, including optic disc, cup/ disc ratio, macula in both eyes using a direct ophthalmoscope through an undilated pupil. All participants with a best-corrected visual acuity of less than 6/12 were subsequently dilated, and the fundus re-checked with an indirect ophthalmoscope. A compound solution of tropicamide (1%) was used to obtain a pupil diameter of at least 6 mm. Those deemed at risk of angle-closure (following an oblique flashlight test) were not dilated. Those with the vertical cup: disc ratio ≥0.70 in either eye in the presence of intraocular pressure of ≥97.5 percentile were identified as having glaucoma [13].

Data analysis

Data were analyzed using Microsoft Excel and Epi Info (version 7.1.2.5) after necessary cleaning and logical checks. Age was categorized into two groups: 40–54 years and ≥55 years. We estimated the prevalence of mild, moderate and severe impaired vision and blindness (as described above) with 95% confidence intervals (CI). We presented the main results stratified by four reporting domains: residence location (urban-rural) and sex (men-women). Age adjustment of prevalence estimates was done based on WHO World Population 2000-2020[14].

Factors associated with impaired vision and blindness were checked with 2×2 cross-tabulation. Unadjusted odds ratios were obtained by univariate logistic regression analysis.

For peer review only - http://bmjopen.bmj.com/site/about/guidelines.xhtml

Finally, independent factors associated with impaired vision and blindness were identified using multiple logistic regression. All variable that had a significant relationship (P<0.05) were entered simultaneously into the model. Adjusted odds ratios and their 95% CIs were obtained to check the strengths of the association. At the same time, P values less than 0.05 were also noted for convenience.

RESULTS

We could recruit 6,391 persons out of the targeted 7,200 resulting in a response rate of 88.8%. Among the respondents, 3436 (53.8%) were women (**Table 1**). Men and women were similar in terms of age categories and average (54.3 years with a standard deviation of 11.2 years). Half (50.9%) of them never attended formal school, and one-fifth (21.9%) had above primary education. Women mainly were homemakers (79.2%), but almost half (48.6%) of men were manual workers. More than 6 in 10 (63.6%) were tobacco (smoking or smokeless) users. However, there was hardly anyone with an alcohol drinking habit (1.2%). One-fifth (20.5%) were overweight (body mass index ≥25.0 kg/m^2), 25.4% had hypertension (blood pressure ≥14/90 mmHg or medication), and 7.8% had diabetes mellitus (random blood glucose ≥11.1 mmol/L or on medication for diabetes).

Low vision and blindness

The prevalence of corrected visual acuity by age, sex and residence are given in **Table 2**. Overall, the age-adjusted prevalence of low vision and blindness was 12.1% and 1.0%, respectively. Blindness was higher in those aged 55 years or older (1.8%) compared to the younger people (0.2%). No differences were observed between sexes and residential areas, as indicated by the overlapping 95% CIs.

Factors associated with low vision and blindness

In our sample,(22.9% had had cataract of some form, 1.7% had diabetic retinopathy, 0.8% had glaucoma, 0.8% had corneal diseases, 0.5 had AMD, and 0.4 had eyelid disorders (**Figure 2)**. Cataract's attribution to blindness was the largest among all. Cataract was present in 76.8% of the blind people. Altogether 84.3% of patients of low vision and blind (**Table 3**). Univariate logistic regression indicated a significant relationship of low vision and blindness with age, male sex, cataract, diabetic retinopathy, glaucoma, and AMD. However, multiple logistic regression after adjusting for age and sex showed a significant association, in order of strength, of cataract (odds ratio 17.0, 95% CI 13.7–21.2), AMD (5.2, 2.1–12.7),

For peer review only - http://bmjopen.bmj.com/site/about/guidelines.xhtml

and diabetic retinopathy (2.2, 1.4–3.5) (**Table 3**). Population attributable risk of cataract for blindness was 79.6%.

DISCUSSION

We report here findings of the second national-level survey, done after 13 years of the first national survey[5] done in 2000, that age-adjusted prevalence of blindness in Bangladeshi adults is 0.9% after best possible correction of vision. This estimate is lower than that reported by the first national survey (1.53%)[5]. However, it is important to note that the first survey was done among those aged 30 years or older. Younger people are expected to have a lower burden of blindness. The ageing of the Bangladeshi population is well known because of the demographic transition[15]. Moreover, the national eye care programme intervention might have contributed to this decline in blindness prevalence.

Prevalence:

The prevalence of blindness in Singapore (0.4%)[16], Taiwan (0.6%)[17], Malaysia (0.3%)[18], China (0.3%)[19] and USA (0.5%)[20] is similar to the prevalence we report here. There was a wide variation of prevalence of blindness in Asian countries like Pakistan is 2.7%[21], Mongolia (1.5%)[22], rural Indonesia (2.2%)[23], India (5.3%)[24], Nepal (1.9%)[25], Nigeria (4.2%)[26], and Iran (1.1%)[27]. These variations, however, may be due to differences in the definition of blindness used in the surveys, age composition of the sample, and survey design. Increasing trend of blindness and visual impairment with age in our sample is somewhat similar to surveys done in India[24] and Iran[27]. Unlike our survey, Pakistan reported a higher prevalence in rural population and in females[21]. Malaysia also reported a higher prevalence in women compared to men[18]. Nonetheless, no sex difference was found in Taiwanese population.

Associated factors/causes

We identified cataract, AMD and diabetic retinopathy as the major causes of blindness in our population. Cataract's attribution to blindness was the largest among all. Cataract is the leading cause of blindness worldwide, especially in Asians[4, 16,17,18,19,21,22,23,24,25,27] including Bangladesh[5]. The leading causes of visual impairment in the Taiwanese population are cataract, amblyopia due to uncorrected refractive errors, vitreo-retinal diseases, corneal blindness and diabetic retinopathy[17]. In Singapore across all ethnic groups, cataract was the leading cause of bilateral blindness. Other major causes of blindness included diabetic retinopathy, AMD, glaucoma, corneal opacity, and myopic maculopathy[16]. In Western countries, AMD is the main cause of blindness, especially after the age of 50 years[28].

For peer review only - http://bmjopen.bmj.com/site/about/guidelines.xhtml

Diabetic retinopathy, as we observed, was important factors for blindness in Taiwan[29], many states of India[30, 31, 32]. However, all the comparison we show here are very much dependent on age and sex of the participating subjects, therefore should be interpreted with caution.

Strengths and limitations

This study has its inherent strength that sample has a national representation, which was drawn from the primary sampling units used by the national statistical authority. It was done by employing a multidisciplinary team that included professional enumerators, opticians, ophthalmic nurses and ophthalmologists. The study, on the other hand, has some limitations too. We could not have colour photos of fundus examinations for subsequent validation of findings. Therefore, some degree of uncertainty of AMD and diabetic retinopathy diagnoses cannot be overruled.

Conclusions

This study provides essential information on blindness burden and its prevention in Bangladesh. The age-adjusted prevalence of blindness in Bangladesh is approximately one percent in adults aged 40 years or older. Cataract, AMD, glaucoma and diabetic retinopathy are the major factors for blindness. The attribution of cataract outweighs all others, being responsible for 80% of the preventable causes. Given that national eye care is primarily based in tertiary care hospitals, we recommend strengthening primary and secondary care systems to reach out to most people who need the services. The creation of public awareness for seeking services could broaden the coverage of national eye care.

Collaborators

Our gratitude goes to Professor Deen Mohd. Noorul Huq and Professor Jalal Ahmed for their guidance, and to Dr Mohd. Abdullah Al Mamun for his support.

Acknowledgements

We acknowledge the contribution provided by Drs Masum Habib, Md Abdul Quader, Zahid Ahsan Mennon, Iftekhar Md Munir and Md Shahabuddin for leading the field team for data collection. We thank Ms Khaleda Akter for her assistance in preparing reference list, formatting the document and obtaining the necessary approval for publication.

Author contribution:

For peer review only - http://bmjopen.bmj.com/site/about/guidelines.xhtml

The study was conceptualized by Shawkat Ara Shakoor (SAS), Mustafizur Rahman (MR), AHM Enayet Hussain (AHMEH) and M Mostafa Zaman (MMZ). The literature review was accomplished by Mohammad Moniruzzaman (MM) and Mahfuzur Rahman Bhuiyan (MRB). Study design and sampling were prepared by MMZ, MM, and MRB. The questionnaire was developed and tested by SAS and MRB. The training manual was drafted, and enumerators were trained by MM, MRB and MMZ. All investigators took part in the data collection, supervision and quality assurance measures. Data cleaning and analysis was done by Ferdous Hakim (FH) and MM with the guidance of MMZ. MMZ critically interpreted results. SAS conceptualized prepared the first draft of the manuscript, which was critically reviewed, revised and finalized by MMZ, MM, MRB, and FH. AHMEH is the guarantor of data. MMZ has guided the whole study.

Competing Interests: The authors of this study declare no conflict of interest. The authors alone are responsible for the views expressed in this article, and they do not necessarily represent the views, decisions or policies of the institutions with which they are affiliated.

Funding: The WHO Country Office for Bangladesh provided financial assistance for this study (WHO Reference: 2013/355662-0, Purchase Order: 200843353, Reg. File: BAN-2013-B7-TSA-0001).

Consent to publish: All authors consent to the publication of this manuscript.

Availability of data and materials: Data are available on reasonable request. Please contact Professor M. Mostafa Zaman at zamanm@who.int.

For peer review only

Table 1: Socio-demographic characteristics and relevant risk factors of the respondents, n (%)

Variables	Both (n=6391)	Men (n=2955)	Women (n=3436)
Age group (years)			
<55	3684 (57.6)	1642 (55.6)	2042 (59.4)
≥55	2707 (42.4)	1313 (44.4)	1394 (40.6)
Residence			
Urban	1922 (30.1)	841 (28.5)	1081 (31.5)
Rural	4469 (69.9)	2114 (71.5)	2355 (68.5)
Education			
No formal schooling	3238 (50.9)	1147 (38.9)	2091 (61.1)
Any primary (classes 1–5)	1733 (27.2)	862 (29.3)	871(25.5)
Above primary (classes ≥6)	1397 (21.9)	937 (31.8)	460 (13.4)
Occupation			
Professional employee[†]	1015 (15.9)	886 (30.1)	129 (3.8)
Industrial worker/ Day laborer	1587 (24.9)	1430 (48.6)	157 (4.6)
Homemaker	2716 (42.6)	0 (0.0)	2716 (79.2)
Unemployed/ Retired	901 (14.1)	503 (17.1)	398 (11.6)
Others[‡]	153 (2.4)	124 (4.2)	29 (0.8)
Tobacco use (smoking or smokeless)	4066 (63.6)	2122 (71.8)	1944 (56.6)
Alcohol use, last 30 days	77 (1.2)	69 (2.3)	8 (0.2)
Overweight/ obesity[§]	1300 (20.5)	455 (15.5)	845 (24.7)
Diabetes mellitus[‖]	498 (7.8)	230 (7.8)	268 (7.8)
Hypertension[¶]	1623 (25.4)	689 (23.3)	934 (27.2)

Missing data for education, 23; occupation, 19; current tobacco use, 15; alcohol use in last 30 days, 21; body mass index, 32; diabetes mellitus, 8.

[*] Cut-off based on mean age (54.3 years).

[†] Professional employment: government and private company employee, businessman.

[‡] Others: shop keeper, weaver, driver, beggar, cook, carpenter, and tailor.

[§] Body mass index ≥25Kg/m^2; 1 pregnant woman was excluded.

[‖] Diabetes mellitus: random capillary blood glucose ≥11.1mmol/L and/ or known history of diabetes; 1 pregnant woman was excluded.

[¶] Hypertension: blood pressure ≥140/90 mgHg or on medication for hypertension.

For peer review only - http://bmjopen.bmj.com/site/about/guidelines.xhtml

Table 2: Prevalence of corrected visual acuities, percent (95% confidence interval)

Characteristics	Number (n=6391)	Normal (≥6/12) (n=5628)	Low vision (≥3/60) (n=154)	Blind (<3/60) (n=56)
Age group*, years				
<55	3684	98.1 (97.6–98.6)	1.7 (1.2–2,2)	0.2 (0.01–0.4)
≥55	2707	74.4 (71.4–77.4)	23.8 (20.9–26.7)	1.8 (1.1–2.5)
Sex				
Men	2955	87.2 (85.1–89.3)	12.0 (10.0–14.1)	0.7 (0.4–1.1)
Women	3436	88.8 (87.4–90.2)	10.2 (8.9–11.6)	1.0 (0.6–1.4)
Residence				
Urban	1922	87.7 (85.2–90.3)	11.8 (9.2–14.3)	0.5 (0.2–0.9)
Rural	4469	88.2 (86.3–90.1)	10.8 (8.9–12.6)	1.0 (0.6–1.4)
**Overall**	**6391**	**88.1 (86.5–89.5)**	**11.1 (9.6–12.6)**	**0.9 (0.6–1.2)**
**Overall (age adjusted) ***		**86.9 (85.2–88.6)**	**12.1 (10.5–13.8)**	**1.0 (0.5–1.4)**

* Adjusted for WHO World Population 2000-2020.[14]

For peer review only

For peer review only - http://bmjopen.bmj.com/site/about/guidelines.xhtml

Table 3: Odds ratios of risk factors for impaired vision and blindness after correction in Bangladeshi adults (n=6391)

Factors		Vision categories		Odds ratio (95% confidence interval)	
		Low vision and blind (<6/12) (n=763)	Normal vision (≥6/12) (n=5628)	Unadjusted	Adjusted for age and sex
Age, years	≥55	693 (90.8)	2014 (35.8)	17.8 (13.8–22.9)*	-
(≥55=1, <55=0)	<55	70 (9.2)	3614 (64.2)	1.0	-
Sex	Men	378 (49.5)	2577 (45.8)	1.2 (1.0–1.4)*	-
(man=1, woman=0)	Women	385 (50.5)	3051 (54.2)	1.0	-
Diabetes mellitus†	Yes	64 (8.4)	435 (7.7)	1.1 (0.8–1.4)	1.0 (0.7–1.3)
(yes=1, no=0)	No	698 (91.6)	5186 (92.3)	1.0	1.0
Hypertension	Yes	192 (25.2)	1431 (25.4)	1.0 (0.8–1.2)	0.8 (0.6–0.9)
(yes=1, no=0)	No	571 (74.8)	4197 (74.6)	1.0	1.0
Cataract	Yes	643 (84.3)	822 (14.6)	31.3 (25.4–38.6)*	17.0 (13.7–21.2)*
(yes=1, no=0)	No	120 (15.7)	4806 (85.4)	1.0	1.0
Diabetic retinopathy	Yes	31 (4.1)	80 (1.4)	2.9 (1.9–4.5)*	2.2 (1.4–3.5)*
(yes=1, no=0)	No	732 (95.9)	5548 (98.6)	1.0	1.0
Glaucoma	Yes	13 (1.7)	40 (0.7)	2.4 (1.3–4.5)*	1.4 (0.7–2.7)
(yes=1, no=0)	No	750 (98.3)	5588 (99.3)	1.0	1.0
AMD‡	Yes	12 (1.6)	17 (0.3)	5.3 (2.5–11.1)*	5.2 (2.1–12.7)*
(yes=1, no=0)	No	751 (98.4)	5611 (99.7)	1.0	1.0
Corneal disease	Yes	6 (0.8)	47 (0.8)	0.9 (0.4–2.2)	0.9 (0.4–2.4)
(yes=1, no=0)	No	757 (99.2)	5581 (99.2)	1.0	1.0
Ocular trauma	Yes	3 (0.4)	7 (0.1)	3.2 (0.8–12.3)	3.4 (0.7–16.6)
(yes=1, no=0)	No	760 (99.6)	5621 (99.9)	1.0	1.0
Eye lid disorder	Yes	4 (0.5)	21 (0.4)	1.4 (0.5–4.1)	0.6 (0.2–1.9)
(yes=1, no=0)	No	759 (99.5)	5607 (99.6)	1.0	1.0

† 8 missing values.

‡ AMD: age related macular degeneration.

* P<0.01

For peer review only - http://bmjopen.bmj.com/site/about/guidelines.xhtml

References

[1] West S, Sommer A. Prevention of blindness and priorities for the future. Bulletin of the World Health Organization, 2001[Cited 2020 May 27];79(3):244–248. Available from: https://www.ncbi.nlm.nih.gov/pmc/articles/PMC2566384/pdf/11285670.pdf

[2] Pascolini D, Mariotti SP. Global estimates of visual impairment: 2010. Br J Ophthalmol. 2012 May;96(5):614-618. DOI: 10.1136/bjophthalmol-2011-300539.

[3] World Health Organization. Blindness and vision impairment. Fact Sheet. 2011 [Internet] [Cited 2011 Oct. 8] World Health Organization. Available from: http://www.who.int/blindness/en/

[4] Ministry of Health and family Welfare, National council for Blind. National Eye Care Plan for implementation of vision 2020 in Bangladesh. [Internet] Ministry of Health and family Welfare, Government of the People's Republic of Bangladesh. [Cited 2020 May 27] Available from: http://nec.gov.bd/opr_pdf/national_eye_care_plan.pdf

[5] Dineen BP, Bourne RR, Ali SM, Huq DMN, Johnson GJ. Prevalence and causes of blindness and visual impairment in Bangladeshi adults: Results of the National Blindness and Low Vision Survey of Bangladesh. Br J Ophthalmol [Internet] 2003 Jul;87(7):820-828. DOI: 10.1136/bjo.87.7.820.

[6] Sight Savers. Bangladesh quadruples its eye care budget. [Accessed 28 October 28, 2020]. https://www.sightsavers.org/news/2017/06/bangladesh-eye-care-budget/

[7] Rosser DA, Laidlaw DAH, Murdoch, IE. The development of a "reduced logMAR" visual acuity chart for use in routine clinical practice. Br J Ophthalmol 2001 Apr;85(4):432–6. DOI: 10.1136/bjo.85.4.432.

[8] Kish L. A procedure for objective respondent selection within the household. Journal of the American Statistical Association.1949:44(247):380-387. DOI:10.1080/01621459.1949.10483314.

[9] WHO Programme for the Prevention of Blindness. (1988). Coding instructions for the WHO/PBL eye examination record (Version III).World Health Organization. [Cited 2020 May 27] Available from: https://apps.who.int/iris/handle/10665/67896

[10] Fletcher AE, Ellwein LB, Selvaraj S, Vijaykumar V, Rahmathullah R, Thulasiraj RD. Measurements of vision function and quality of life in patients with cataracts in southern India. Arch Ophthalmol. 1997 Jun;115(6):767–774. DOI: 10.1001/archopht.1997.01100150769013

[11] World Health Organization. Blindness and vision impairment: Definitions. [Internet]. Available from: https://www.who.int/news-room/fact-sheets/detail/blindness-and-visual-impairment [accessed: 17 June 2020]

[12] Dunn G. Design and analysis of reliability studies: the statistical evaluation of measurement errors. London: Edward Arnold; New York: Oxford University Press, 1989.

[13] Paul J Foster, Ralf Buhrmann, Harry A Quigley, Gordon J Johnson. The definition and classification of glaucoma in prevalence surveys. Br J Ophthalmol 2002; DOI: 10.1136/bjo.86.2.238

For peer review only - http://bmjopen.bmj.com/site/about/guidelines.xhtml

[14] Ahmad OB, Boschi-Pinto C, Lopez AD, et al. Age Standardization of Rates: A New WHO Standard. GPE Discussion Paper Series: No31 Geneva, Switzerland: World Health Organization, 2001.

[15] Gaur A. Demographic Transition of Bangladesh. Int J Science Research 2018; DOI: 10.21275/ART20203387

[16] Wong TY, Zheng Y, Wong WL, Lamoureux EL, Wang JJ, Mitchell P, Cheung N, Aung T, Saw SM, Cheng CY. The Prevalence and Causes of Visual Impairment and Blindness in a Multi-Ethnic Asian Population: The Singapore Epidemiology of Eye Disease (SEED) Study [Internet]. Invest Ophthalmol & Vis Scien. 2012 Mar, 53(14), 5640. (DOI: Not Available) Available from: https://iovs.arvojournals.org/article.aspx?articleid=2359339

[17] Liu JH, Cheng CY, Chen SJ, Lee FL. Visual Impairment in a Taiwanese Population: Prevalence, Causes, and Socioeconomic Factors. Ophthalmic Epidemiol. 2001 Dec;8(5):339-50, DOI:10.1080/09286586.2001.11644261.

[18] Zainal M, Ismail SM, Ropilah AR, Elias H, Arumugam G, Alias D, Fathilah J, Lim TO, Ding LM, Goh PP. Prevalence of Blindness and Low Vision in Malaysian Population: Results From the National Eye Survey 1996. Br J Ophthalmol. 2002 Sep; 86(9): 951–95. DOI: 10.1136/bjo.86.9.951.

[19] Xu L, Wang Y, Li Y, Wang Y, Cui T, Li J, Jonas JB. Causes of Blindness and Visual Impairment in Urban and Rural Areas in Beijing. Ophthalmol. 2006 Jul;113(7):1134.e1-1134.e11. DOI: 10.1016/j.ophtha.2006.01.035

[20] Bourne RRA, Flaxman SR, Braithwaite T, Cicinelli MV, Das A, Jonas JB, Keeffe J, Kempen JH, Leasher J, Limburg H, Naidoo K, Pesudovs K, Resnikoff S, Silvester A, Stevens GA, Tahhan N, Wong TY, Taylor HR, on behalf of the Vision Loss Expert Group. Magnitude, temporal trends, and projections of the global prevalence of blindness and distance and near vision impairment: a systematic review and meta-analysis. Lancet Glob Health 2017 Sep; 5(9):e888–e897. DOI: 10.1016/S2214-109X(17)30293-0.

[21] Jadoon MZ, Dineen B, Bourne RR, Shah SP, Khan MA, Johnson GJ, Gilbert CE, Khan MD, on behalf of the Pakistan National Eye Survey Study Group. Prevalence of Blindness and Visual Impairment in Pakistan: The Pakistan National Blindness and Visual Impairment Survey. Invest Ophthalmol Vis Sci, 2006 Nov:47(11):4749-4755. DOI: 10.1167/iovs.06-0374.

[22] Baasanhu J, Johnson GJ, Burendei G, Minassian DC. Prevalence and causes of blindness and visual impairment in Mongolia: A survey of populations aged 40 years and older. Bull World Health Organ. 1994:72(5):771-776. Available from: https://www.ncbi.nlm.nih.gov/pmc/articles/PMC2486559/pdf/bullwho00416-0077.pdf

[23] Saw SM, Husain R, Gazzard GM, Koh D, Widjaja D, Tan DTH. Causes of low vision and blindness in rural Indonesia. Br J Ophthalmol. 2003 Oct; 87(9):1075-1078. DOI: 10.1136/bjo.87.9.1075

[24] Murthy GVS, Gupta SK, Bachani D, Jose R, John N. Current estimates of blindness in India. Br J Ophthalmol. 2005 Mar; 89(3):257–260. DOI: 10.1136/bjo.2004.056937

[25] Thapa R, Bajimaya S, Paudyal G, Khanal S, Tan S, Thapa SS, Van Rens GHMB. Prevalence and causes of low vision and blindness in an elderly population in Nepal: the

For peer review only - http://bmjopen.bmj.com/site/about/guidelines.xhtml

Bhaktapur retina study. BMC Ophthalmol. 2018 Feb;18:42. DOI: 10.1186/s12886-018-0710-9

[26] International Centre for Eye Health, Institute of Ophthalmology London, National Programme for the Prevention of Blindness Federal Ministry of Health Nigeria, National Eye Centre Nigeria, Sightsavers International Nigeria. The Nigeria national blindness and visual impairment survey 2005-2007. Available from: blogs.lshtm.ac.uk/iceh/files/2014/04/NigeriaSurvey.pdf

[27] Soori H, Ali JM, Nasrin R. Prevalence and Causes of Low Vision and Blindness in Tehran Province, Iran. J Pak Med Assoc.[Internet] 2011 Jun:61(6):544-549. Available from: https://jpma.org.pk/article-details/2818?article_id=2818

[28] National Institute of Health. National Eye Institute. Age-related macular degeneration. [Accessed 31 October 2020]. https://www.nei.nih.gov/learn-about-eye-health/eye-conditions-and-diseases/age-related-macular-degeneration

[29] Chen MS, Kao CS, Chang CJ, Wu TJ, Fu CC, Chen CJ, Tai TY. Prevalence and risk factors of diabetic retinopathy among noninsulin-dependent diabetic subjects. Am J Ophthalmol. 1992 Dec;114(6):723-730. DOI: 10.1016/s0002-9394(14)74051-6.

[30] Dandona L, Dandona R, Naduvilath TJ, McCarty CA, Rao GN. Population based assessment of diabetic retinopathy in an urban population in southern India. Br J Ophthalmol. 1999 Aug; 83(8):937-940. DOI: 10.1136/bjo.83.8.937.

[31] Nirmalan PK, Katz J, Robin AL, Tielsch JM, Namperumalsamy P, Kim R, Narendran V, Ramakrishnan R, Krishnadas R, Thulasiraj RD, Suan Eric. Prevalence of Vitreoretinal Disorders in a Rural Population of Southern India: The Aravind Comprehensive Eye Study. Arch Ophthalmol. 2004 Apr;122(4)581-586. DOI: 10.1001/archopht.122.4.581.

[32] Wong TY, Loon SC, Saw SW. The epidemiology of age-related eye diseases in Asia. Br J Ophthalmol 2006 Apr;90(4):506-511. DOI: 10.1136/bjo.2005.083733

For peer review only

Figure 1: Flowchart for subject selection of the cross-sectional national survey done in urban and rural areas of all seven divisions in Bangladesh (n=6391)

.
*HH indicates household; **PSU, primary sampling unit.

Figure 2. Prevalence of various eye conditions among the respondents of the cross-sectional national survey on visual impairments in Bangladesh (n=6391)

For peer review only

For peer review only - http://bmjopen.bmj.com/site/about/guidelines.xhtml

Figure 1. Selection of subjects aged ≥ 40 years and data collection for the blindness survey in Bangladesh

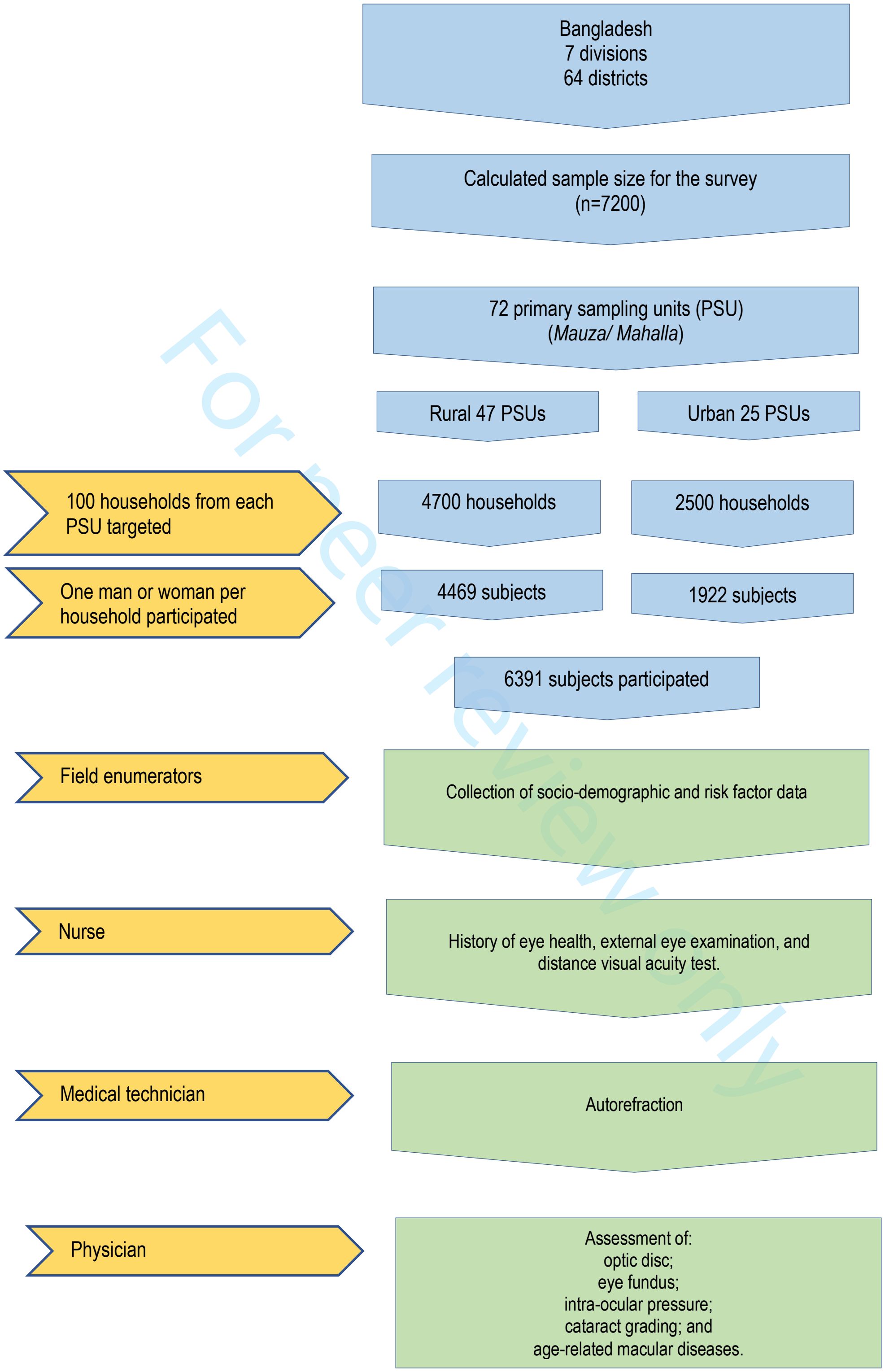

For peer review only

Figure 2

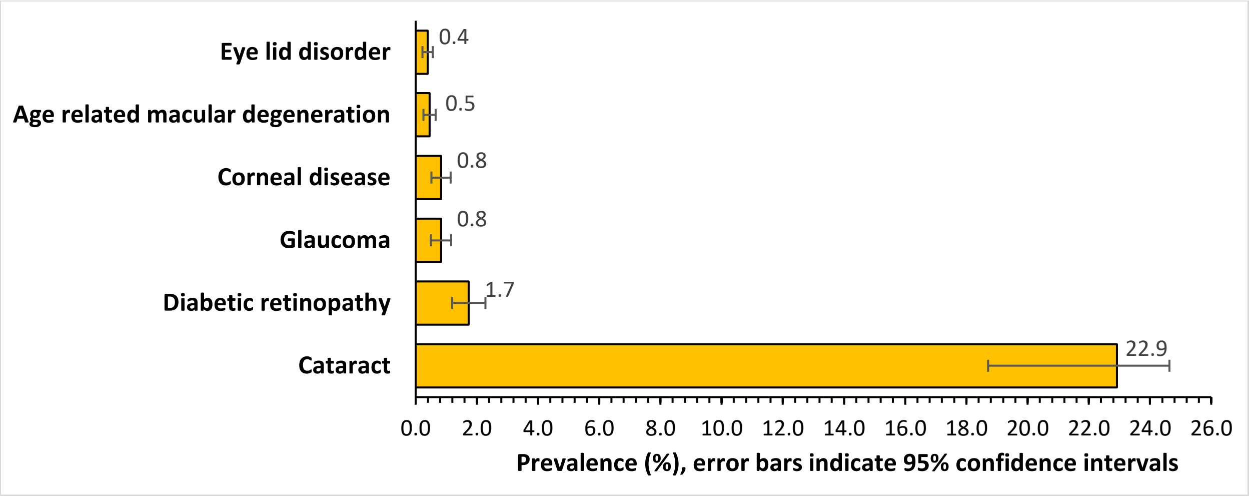

peer review only

STROBE (Strengthening The Reporting of OBservational Studies in Epidemiology) Checklist

A checklist of items that should be included in reports of observational studies. You must report the page number in your manuscript where you consider each of the items listed in this checklist. If you have not included this information, either revise your manuscript accordingly before submitting or note N/A.

Note: An Explanation and Elaboration article discusses each checklist item and gives methodological background and published examples of transparent reporting. The STROBE checklist is best used in conjunction with this article (freely available on the Web sites of PLoS Medicine at http://www.plosmedicine.org/, Annals of Internal Medicine at http://www.annals.org/, and Epidemiology at http://www.epidem.com/). Information on the STROBE Initiative is available at www.strobe-statement.org.

Section and Item	Item No.	Recommendation	Reported on Page No.
**Title and Abstract**	1	(*a*) Indicate the study's design with a commonly used term in the title or the abstract	
		(*b*) Provide in the abstract an informative and balanced summary of what was done and what was found	
**Introduction**			
Background/Rationale	2	Explain the scientific background and rationale for the investigation being reported	
Objectives	3	State specific objectives, including any prespecified hypotheses	
**Methods**			
Study Design	4	Present key elements of study design early in the paper	
Setting	5	Describe the setting, locations, and relevant dates, including periods of recruitment, exposure, follow-up, and data collection	
Participants	6	(*a*) *Cohort study*—Give the eligibility criteria, and the sources and methods of selection of participants. Describe methods of follow-up<br>*Case-control study*—Give the eligibility criteria, and the sources and methods of case ascertainment and control selection. Give the rationale for the choice of cases and controls<br>*Cross-sectional study*—Give the eligibility criteria, and the sources and methods of selection of participants	
		(*b*) *Cohort study*—For matched studies, give matching criteria and number of exposed and unexposed<br>*Case-control study*—For matched studies, give matching criteria and the number of controls per case	
Variables	7	Clearly define all outcomes, exposures, predictors, potential confounders, and effect modifiers. Give diagnostic criteria, if applicable	

For peer review only

Section and Item	Item No.	Recommendation	Reported on Page No.
Data Sources/ Measurement	8*	For each variable of interest, give sources of data and details of methods of assessment (measurement). Describe comparability of assessment methods if there is more than one group	
Bias	9	Describe any efforts to address potential sources of bias	
Study Size	10	Explain how the study size was arrived at	
Quantitative Variables	11	Explain how quantitative variables were handled in the analyses. If applicable, describe which groupings were chosen and why	
Statistical Methods	12	(*a*) Describe all statistical methods, including those used to control for confounding	
		(*b*) Describe any methods used to examine subgroups and interactions	
		(*c*) Explain how missing data were addressed	
		(*d*) *Cohort study*—If applicable, explain how loss to follow-up was addressed<br>*Case-control study*—If applicable, explain how matching of cases and controls was addressed<br>*Cross-sectional study*—If applicable, describe analytical methods taking account of sampling strategy	
		(*e*) Describe any sensitivity analyses	
**Results**			
Participants	13*	(a) Report numbers of individuals at each stage of study—eg numbers potentially eligible, examined for eligibility, confirmed eligible, included in the study, completing follow-up, and analysed	
		(b) Give reasons for non-participation at each stage	
		(c) Consider use of a flow diagram	
Descriptive Data	14*	(a) Give characteristics of study participants (eg demographic, clinical, social) and information on exposures and potential confounders	
		(b) Indicate number of participants with missing data for each variable of interest	
		(c) *Cohort study*—Summarise follow-up time (eg, average and total amount)	
Outcome Data	15*	*Cohort study*—Report numbers of outcome events or summary measures over time	
		Case-control study—Report numbers in each exposure category, or summary measures of exposure	
		Cross-sectional study—Report numbers of outcome events or summary measures	

For peer review only

Section and Item	Item No.	Recommendation	Reported on Page No.
Main Results	16	(*a*) Give unadjusted estimates and, if applicable, confounder-adjusted estimates and their precision (eg, 95% confidence interval). Make clear which confounders were adjusted for and why they were included	
		(*b*) Report category boundaries when continuous variables were categorized	
		(*c*) If relevant, consider translating estimates of relative risk into absolute risk for a meaningful time period	
Other Analyses	17	Report other analyses done—eg analyses of subgroups and interactions, and sensitivity analyses	
**Discussion**			
Key Results	18	Summarise key results with reference to study objectives	
Limitations	19	Discuss limitations of the study, taking into account sources of potential bias or imprecision. Discuss both direction and magnitude of any potential bias	
Interpretation	20	Give a cautious overall interpretation of results considering objectives, limitations, multiplicity of analyses, results from similar studies, and other relevant evidence	
Generalisability	21	Discuss the generalisability (external validity) of the study results	
**Other Information**			
Funding	22	Give the source of funding and the role of the funders for the present study and, if applicable, for the original study on which the present article is based	

*Give information separately for cases and controls in case-control studies and, if applicable, for exposed and unexposed groups in cohort and cross-sectional studies.

Once you have completed this checklist, please save a copy and upload it as part of your submission. DO NOT include this checklist as part of the main manuscript document. It must be uploaded as a separate file.

For peer review only

Prevalence of blindness and its determinants in Bangladeshi adult population: Results from a national cross-sectional survey

Journal:	*BMJ Open*
Manuscript ID	bmjopen-2021-052247.R1
Article Type:	Original research
Date Submitted by the Author:	22-Nov-2021
Complete List of Authors:	Ara Shakoor, Shawkat; National Institute of Opthalmology<br>Rahman, Mustafizur ; Dhaka Medical College and Hospital<br>Hossain, A.H.M. Enayet; National Institute of Opthalmology<br>Moniruzzaman, Mohammad; Shiga University of Medical Science, Public Health<br>Bhuiyan, Mahfuzur ; National Heart Foundation of Bangladesh<br>Hakim, Ferdous; World Health Organization Bangladesh, Research and Publication<br>Zaman, MM; World Health Organization Bangladesh, Research and Publication
**Primary Subject Heading**:	Ophthalmology
Secondary Subject Heading:	Epidemiology, Public health
Keywords:	Cataract and refractive surgery < OPHTHALMOLOGY, Glaucoma < OPHTHALMOLOGY, EPIDEMIOLOGY, OPHTHALMOLOGY, Corneal and external diseases < OPHTHALMOLOGY

I, the Submitting Author has the right to grant and does grant on behalf of all authors of the Work (as defined in the below author licence), an exclusive licence and/or a non-exclusive licence for contributions from authors who are: i) UK Crown employees; ii) where BMJ has agreed a CC-BY licence shall apply, and/or iii) in accordance with the terms applicable for US Federal Government officers or employees acting as part of their official duties; on a worldwide, perpetual, irrevocable, royalty-free basis to BMJ Publishing Group Ltd ("BMJ") its licensees and where the relevant Journal is co-owned by BMJ to the co-owners of the Journal, to publish the Work in this journal and any other BMJ products and to exploit all rights, as set out in our licence.

The Submitting Author accepts and understands that any supply made under these terms is made by BMJ to the Submitting Author unless you are acting as an employee on behalf of your employer or a postgraduate student of an affiliated institution which is paying any applicable article publishing charge ("APC") for Open Access articles. Where the Submitting Author wishes to make the Work available on an Open Access basis (and intends to pay the relevant APC), the terms of reuse of such Open Access shall be governed by a Creative Commons licence – details of these licences and which Creative Commons licence will apply to this Work are set out in our licence referred to above.

Other than as permitted in any relevant BMJ Author's Self Archiving Policies, I confirm this Work has not been accepted for publication elsewhere, is not being considered for publication elsewhere and does not duplicate material already published. I confirm all authors consent to publication of this Work and authorise the granting of this licence.

For peer review only

Prevalence of blindness and its determinants in Bangladeshi adult population: Results from a national cross-sectional survey

Shawkat Ara Shakoor[1], MScCEH, MS; ORCID: https://orcid.org/0000-0001-6299-7529

Mustafizur Rahman[2], MBBS; MS; ORCID: https://orcid.org/0000-0002-7199-1070

A H M Enayet Hussain[3], FCPS, FRCS; ORCID: https://orcid.org/0000-0003-2715-5574

Mohammad Moniruzzaman[4], MPhil, PhD; ORCID: https://orcid.org/0000-0003-2144-7111;

Mahfuzur Rahman Bhuiyan[5], MBBS, MPH; ORCID: https://orcid.org/0000-0001-6962-7264;

Ferdous Hakim[6], MBBS, MPH; ORCID: https://orcid.org/0000-0003-2376-3978;

M Mostafa Zaman[7], MBBS, PhD; ORCID: https://orcid.org/0000-0002-1736-1342

Affiliations:

[1]National Institute of Ophthalmology, Dhaka, Bangladesh; Email: millyshakoor65@gmail.com

[2]Dhaka Medical College, Dhaka, Bangladesh. Email: mdmustafizur.ophth@gmail.com

[3]Directorate General of Health Services, Dhaka, Bangladesh; Email: paedeye@yahoo.com

[4]Shiga University of Medical Science, Shiga, Japan; Email: mmzbd82@gmail.com

[5]National Heart Foundation of Bangladesh, Dhaka, Bangladesh; Email: mahfuzdoc@yahoo.com

[6, 7]World Health Organization, Dhaka, Bangladesh; Email: hakimf@who.int; zamanm@who.int

Running head: Blindness in Bangladeshi adults

Word count: Main text: 2495, Abstract: 297

Tables: 3

Figures: 3

Address for correspondence:

Professor Dr M Mostafa Zaman, Adviser, Research and Publication, World Health Organization, 10 Gulshan Avenue, Road Number 5, Gulshan 1, Dhaka 1212, Bangladesh. Phone: +88028831415, Mobile: +8801714165205, Fax: +88028831423, Email: zamanm@who.int, mmostafazaman@gmail.com

For peer review only

For peer review only - http://bmjopen.bmj.com/site/about/guidelines.xhtml

ABSTRACT

Objective:

The objective of this study was to determine the prevalence of blindness and its determinants in Bangladeshi adult population.

Study design:

A cross-sectional population-based survey conducted at household level with national representation. Samples were drawn from the 2011 national census frame using a multistage stratified cluster sampling method.

Setting and participants:

The survey was done in urban and rural areas in 2013 using a probability proportionate to size sampling approach to locate participants from 72 primary sampling units. One man or one woman aged ≥40 years was randomly selected from their households to recruit 7,200. In addition to socio-demographic data, information on medication for hypertension and diabetes was obtained. Blood pressure and capillary blood glucose were measured. Eyelids, cornea, lens, and retina were examined in addition to visual acuity and refraction testing.

Primary outcome measures:

The following definition was used to categorize subjects having: (a) blindness: visual acuity <3/60, (b) low vision: ≥3/60–<6/60, and (c) normal vision: ≥6/12 after best correction.

Results:

We could recruit 6,391 (88.8%) people among whom, 2955 (46,2%) were men. Among them, 1922 (30.1%) were from urban and 4469 (69.9%) from rural areas. The mean (standard deviation) age was 54.3 (11.2) years. The age-standardized prevalence, after best correction, of blindness and low vision was 1.0% (95% confidence interval, 0.5–1.4) and 12.1% (10.5–13.8) respectively. Multivariable logistic regression indicated that cataract, age-related macular degeneration, and diabetic retinopathy were significantly associated with low vision and blindness after adjustment for age and sex. Population attributable risk of cataract for low vision and blindness was 79.6%.

Conclusion:

Low vision and blindness are common problems in those aged 40 years or older. Extensive screening, and eye care services are necessary for wider coverage engaging all tiers of the health care system especially focusing on cataract.

Key words: Bangladesh, Adults, Population, Low vision, Blindness

For peer review only

Article Summary

Strengths and limitations of this study

- This nationally representative population-based survey indicates that more than 1 in 10 Bangleashi adults aged ≥40 years have low vision or blindness; cataract being the single most attributing factor.
- The study followed rigorous survey methods, including a multistage, geographically clustered, and probability proportional to size sampling approach to recruit particiapnts randomly.
- The absence of colour photos of fundus examinations might have led to biased estimate of age-related macular degeneration and diabetic retinopathy.

For peer review only

For peer review only - http://bmjopen.bmj.com/site/about/guidelines.xhtml

BACKGROUND

The impact of visual loss on an individual's personal, economic, and social life is profound. When the burden of blindness in communities is high, the consequences become a significant public health issue.[1] According to the World Health Organization (WHO), 285 million people globally lived with visual impairment in 2010. Of them, 246 million had low vision, 39 million were blind, and two-thirds of this population were aged over 50 years.[2] Because of the rapid population ageing, low vision and blindness have become a global public health threat, particularly in developing countries.

Nearly 90% of the world's visually impaired people live in developing countries. The South-East Asia Region, including Bangladesh, is estimated to inhabit 90.5 million visually impaired and 12 million blind adults in 2010.[3] Globally the top four causes of visual impairment are uncorrected refractive errors, cataract, age-related macular degeneration (AMD), and glaucoma. Therefore, 80% of all visual impairments are avoidable.[3]

In Bangladesh, a previous national survey—done in 2000—reported an age-standardized prevalence of blindness and low vision of 1.53% and 0.56%, respectively, among adults aged 30 years or older.[4, 5] Since then, Bangladesh has passed through a remarkable demographic transition. Recent data on blindness and low vision in Bangladesh are unknown. Bangladesh has been implementing its National Eye Care for preventing avoidable blindness and low vision, but mostly through tertiary level hospitals.[6] A recent estimate, therefore, was required to inform the eye care plan and other relevant programmes. We conducted this national survey to determine the prevalence of blindness and impaired vision, and related factors in Bangladeshi adults.

For peer review only

METHODS

Study design, population, and setting

We conducted a nationwide population-based cross-sectional survey among Bangladeshi adults (men and women) aged 40 years or older in September—December 2013. We calculated our sample size based on a prevalence of blindness (1.53%), with a margin of error (0.00765) and a design effect of 1.5 (1483). Then we adjusted for four groups (men, women, urban and rural) and a response rate of 82.5% (7193), leaving the final sample size to 7200. The details of the sampling procedure have been described previously.[7] Briefly, we adopted a multistage, geographically clustered, probability-based sampling approach to obtain a nationally representative sample. We invited a total of 7,200 randomly selected

For peer review only - http://bmjopen.bmj.com/site/about/guidelines.xhtml

adults from 72 (urban, 25; rural, 47) primary sampling units (used in the 2011 national census) to participate from all seven divisions of Bangladesh. In each selected primary sampling unit, we identified 100 consecutive households with a random start. Then we randomly selected one participant from a list of eligible household members using the Kish table.[8] The flowchart of subject selection is given in **Figure 1.**

Patient and Public involvement

Patient and public were not involved in this study.

Training of the survey team

The survey team was comprised of experienced enumerators, ophthalmic nurses, medical technologists and ophthalmologists. They were trained in the National Institute of Ophthalmology by the investigators. Upon completion of their training, a dry-run was given in two nearby rural and urban areas. They were trained (as a team) using a using a study manual before launching the survey to reduce inter-observer variations and improve diagnostic accuracy. Their findings were randomly checked by the investigators at least once in each primary sampling unit.

Data collection

As depicted in **Figure 1**, trained enumerators collected demographic, socio-economic, and medical history data using an interviewer-administered standardized questionnaire at the household level. Thereafter, they invited participants to have a physical and ophthalmic examination in a nearby health centre (or make-shift examination centre established conveniently by the research team). Nurses measured participants' height, weight, seated blood pressure, and (random) capillary blood glucose using a glucometer (Accu-chek Advantage, Roche Diagnostics Division, Switzerland). We used a modified WHO/PBL questionnaire Version III[9] as our instruments.[10]

Ethics approval

We obtained ethical approval for this study from the Institutional Review Board of the National Institute of Ophthalmology, Dhaka, Bangladesh (Memo No. NIO/670 of 4 April 2013). All participants gave written consent through signature, if not possible, through thumbprint.

For peer review only - http://bmjopen.bmj.com/site/about/guidelines.xhtml

Vision and ophthalmic examinations

We used WHO International Classification of Diseases 10 categories of visual impairment for the study.[11, 12] Blindness was defined as corrected visual acuity of less than 3/60 in the better eye. Low vision was defined as corrected visual acuity of less than 6/60 but equal to or more than 3/60 in the better eye. People having visual acuity of 6/12 or more were considered to have normal vision.

Eye lids, cornea, lens (including its absence or displacement) and retina were examined. Age-related macular degeneration (AMD) was defined as the presence of any one of the following: soft drusen or reticular drusen, hyper- or hypopigmentation of the retinal pigment epithelium. Diabetic retinopathy included non-proliferative, proliferative, and maculopathy subtypes. These were not mutually exclusive, as the latter two types, for example, may co-exist.

Ophthalmic nurses examined blood pressure, capillary blood glucose and took medical history of diseases such as hypertension and diabetes. Hypertension was defined as blood pressure ≥140/90 mm Hg or use of antihypertensive medicines, and diabetes was defined as casual capillary blood glucose ≥11.1 mmol/dL or use of antidiabetic medicines. Distance visual acuity was measured on unaided participants with Snellen 'E' chart and a hand-held tally counter, if necessary, at three meters by ophthalmic nurses. Depending on acuity, finger count, hand movement and light projections were used. Medical technologists have done autorefraction. Thereafter, subjective refractions were done by the ophthalmologists. Based on presenting visual acuity, participants were assigned either a red card (acuity worse than 6/12 in either eye) or a green card (equal or better than 6/12 in both eyes tested separately).

Intra-ocular pressure was measured using Schiotz tonometer after application of Tetracaine hydrochloride (1%). A relative afferent pupil defect in those patients with a best-corrected visual acuity of <6/12 in either eye was tested. The ophthalmologist assessed the fundus, including optic disc, cup/ disc ratio, macula in both eyes using a direct ophthalmoscope through an undilated pupil. All participants with a best-corrected visual acuity of less than 6/12 were subsequently dilated, and the fundus re-checked with an indirect ophthalmoscope. A compound solution of tropicamide (1%) was used to obtain a pupil diameter of at least 6 mm. Those deemed at risk of angle-closure (following an oblique flashlight test) were not

For peer review only - http://bmjopen.bmj.com/site/about/guidelines.xhtml

dilated. Those with the vertical cup: disc ratio ≥0.70 in either eye in the presence of intraocular pressure of ≥97.5 percentile were identified as having glaucoma.[13]

Data analysis

Data were analyzed using Microsoft Excel and Epi Info (version 7.1.2.5) after necessary cleaning and logical checks. Age was categorized into two groups: 40–54 years and ≥55 years. We estimated the prevalence of mild, moderate and severe impaired vision and blindness (as described above) with 95% confidence intervals (CI). We presented the main results stratified by four reporting domains: residence location (urban-rural) and sex (men-women). Age adjustment of prevalence estimates was done based on WHO World Population 2000-2020.[14]

Factors associated with impaired vision and blindness were checked with 2×2 cross-tabulation. Unadjusted odds ratios were obtained by univariate logistic regression analysis. Finally, risk factors independent of age and sex were identified using multiple logistic regression. Age and sex were entered into all the models. Thus, adjusted odds ratios and their 95% CIs were obtained to check the strengths of the association. At the same time, P values less than 0.05 were also noted for convenience.

RESULTS

We could recruit 6,391 persons out of the targeted 7,200 resulting in a response rate of 88.8%. Among the respondents, 3436 (53.8%) were women (**Table 1**). Men and women were similar in terms of age categories and average (54.3 years with a standard deviation of 11.2 years). Half (50.9%) of them never attended formal school, and one-fifth (21.9%) had above primary education. Women mainly were homemakers (79.2%), but almost half (48.6%) of men were manual workers. More than 6 in 10 (63.6%) were tobacco (smoking or smokeless) users. However, there was hardly anyone with an alcohol drinking habit (1.2%). One-fifth (20.5%) were overweight (body mass index ≥25.0 kg/m^2), 25.4% had hypertension (blood pressure ≥14/90 mmHg or medication), and 7.8% had diabetes mellitus (random blood glucose ≥11.1 mmol/L or on medication for diabetes).

Low vision and blindness

The prevalence of corrected visual acuity by age, sex and residence are given in **Table 2**. Overall, the age-adjusted prevalence of low vision and blindness was 12.1% and 1.0%, respectively. Blindness was higher in those aged 55 years or older (1.8%) compared to the

For peer review only - http://bmjopen.bmj.com/site/about/guidelines.xhtml

younger people (0.2%) (<55 years old). Further splitting of age showed an increasing trend of blindness prevalence across age groups (**Figure 2**). No differences were observed between sexes and residential areas, as indicated by the overlapping 95% CIs (**Table 2)**.

Factors associated with low vision and blindness

In our sample, 22.9% (95% CI, 18.7–24.6%) had had cataract of some form, 1.7% (1.2–2.3%) had diabetic retinopathy, 0.8% (0.5–1.2%) had glaucoma, 0.8% (0.5–1.1%) had corneal diseases, 0.5 (0.3–0.7%) had AMD, and 0.4 (0.2–0.6%) had eyelid disorders (**Figure 3)**. Altogether 84.3% of patients with low vision and blindness had cataract (**Table 3**). Univariate logistic regression indicated a significant relationship of low vision and blindness with age, male sex, cataract, diabetic retinopathy, glaucoma, and AMD. However, multiple logistic regression after adjusting for age and sex showed a significant association, in order of strength, of cataract (odds ratio 17.0, 95% CI 13.7–21.2), AMD (5.2, 2.1–12.7), and diabetic retinopathy (2.2, 1.4–3.5) (**Table 3**). Cataract's attribution to blindness was the largest among all. Population attributable risk of cataract for blindness was 79.6%.

DISCUSSION

We report here findings of the second national-level survey, done after 13 years of the first national survey[5] done in 2000, that age-adjusted prevalence of blindness in Bangladeshi adults is 1.0% after best possible correction of vision. This estimate is lower than that reported by the first national survey (1.53%).[5] However, it is important to note that the first survey was done among those aged 30 years or older. Younger people are expected to have a lower burden of blindness. The ageing of the Bangladeshi population is well known because of the demographic transition.[15] Moreover, the national eye care programme intervention might have contributed to this decline in blindness prevalence. The national eye care plan[4] emphasized activities to reduce blindness focusing cataract surgery that is low-cost, organizing outreach camps for screening, awareness creation, and manpower training. The plan facilitated establishment of treatment centers at district level, and eyesight testing through partnership of government and non-governmental organizations.

<u>Prevalence</u>:

The prevalence of blindness in Singapore (0.4%)[16], Taiwan (0.6%)[17], Malaysia (0.3%)[18], China (0.3%)[19] and USA (0.5%)[20] is similar to the prevalence we report here (1.0%). There was a wide variation of prevalence of blindness in Asian countries like Pakistan is 2.7%[21], Mongolia (1.5%)[22], rural Indonesia (2.2%)[23], India (5.3%)[24],

For peer review only - http://bmjopen.bmj.com/site/about/guidelines.xhtml

Nepal (1.9%)[25], Nigeria (4.2%)[26], and Iran (1.1%).[27] These variations, however, may be due to differences in the definition of blindness used in the surveys, age composition of the sample, and survey design. Increasing trend of blindness and visual impairment with age in our sample is somewhat similar to surveys done in India[24] and Iran.[27] Unlike our survey, Pakistan reported a higher prevalence in rural population and in females.[21] Malaysia also reported a higher prevalence in women compared to men.[18] Nonetheless, no sex difference was found in Taiwanese population.

Apparently, we observed a higher prevalence of low vision (12.1%) compared to studies in India (9.3%)[24], Pakistan (3.3)[21], Iran (4.0%)[27], but it was somewhat similar to that reported from South American countries (5.9 --18.7%).[28] These differences should be cautiously interpreted because variation in age composition of the respondents, and some other factors, is an important determinant of low vision.

Associated factors/causes

We identified cataract, AMD and diabetic retinopathy as the major causes of blindness in our population. Cataract's attribution to blindness was the largest among all. Cataract is the leading cause of blindness worldwide, and responsible for 94 million blindness.[3] This is true for Asians countries.[4, 16-19, 21-25, 27] including Bangladesh.[5] The leading causes of visual impairment in the Taiwanese population are cataract, amblyopia due to uncorrected refractive errors, vitreo-retinal diseases, corneal blindness and diabetic retinopathy.[17] In Singapore across all ethnic groups, cataract was the leading cause of bilateral blindness. Other major causes of blindness included diabetic retinopathy, AMD, glaucoma, corneal opacity, and myopic maculopathy.[16] In Western countries, AMD is the main cause of blindness, especially after the age of 50 years.[29] Diabetic retinopathy, as we observed, was important factors for blindness in Taiwan[30], many states of India.[31-33] However, all the comparison we show here are very much dependent on age and sex of the participating subjects, therefore should be interpreted with caution.

Cataracts attribution to blindness in our sample (79.6%) is a little higher than that reported in an India population (62.1%).[34] Therefore, addressing cataract will be bring most benefit to prevent blindness. In addition to promotion of healthy ageing, a few other factors such as ultraviolet ray exposures, diabetes, hypertension, use of certain drugs, and smoking can be considered.[35, 36] Accessibility to socioeconomically deprived people especially in remote areas should be enhanced. Blindness prevention programe's success will largely depend on

For peer review only - http://bmjopen.bmj.com/site/about/guidelines.xhtml

the health system's capacity building to deliver low-cost cataract surgeries. Supplementation from outreach screening will be valuable.

Strengths and limitations

This study has its inherent strength that sample has a national representation, which was drawn from the primary sampling units used by the national statistical authority. It was done by employing a multidisciplinary team that included professional enumerators, ophthalmic nurses, medical technologists, and ophthalmologists. The study, on the other hand, has some limitations too. We could not have colour photos of fundus examinations for subsequent validation of findings. Therefore, some degree of underestimation of AMD and diabetic retinopathy diagnoses cannot be overruled.

Conclusions

This study provides essential information on blindness burden and its prevention in Bangladesh. The age-adjusted prevalence of blindness in Bangladesh is approximately one percent in adults aged 40 years or older. Cataract, AMD, glaucoma and diabetic retinopathy are the major factors for blindness. The attribution of cataract outweighs all others, being responsible for 80% of the preventable causes. Given that national eye care is primarily based in tertiary care hospitals, we recommend strengthening primary and secondary care systems to reach out to most people who need the services. The creation of public awareness for seeking services could broaden the coverage of national eye care.

Collaborators

Our gratitude goes to Professor Deen Mohd. Noorul Huq and Professor Jalal Ahmed for their guidance, and to Dr Mohd. Abdullah Al Mamun for his support.

Acknowledgements

We acknowledge the contribution provided by Drs Masum Habib, Md Abdul Quader, Zahid Ahsan Mennon, Iftekhar Md Munir and Md Shahabuddin for leading the field team for data collection. We thank Ms Khaleda Akter for her assistance in preparing reference list, formatting the document and obtaining the necessary approval for publication.

Author contribution:

The study was conceptualized by Shawkat Ara Shakoor (SAS), Mustafizur Rahman (MR), AHM Enayet Hussain (AHMEH) and M Mostafa Zaman (MMZ). The literature review was

For peer review only - http://bmjopen.bmj.com/site/about/guidelines.xhtml

accomplished by Mohammad Moniruzzaman (MM) and Mahfuzur Rahman Bhuiyan (MRB). Study design and sampling were prepared by MMZ, MM, and MRB. The questionnaire was developed and tested by SAS and MRB. The training manual was drafted, and enumerators were trained by MM, MRB and MMZ. All investigators took part in the data collection, supervision and quality assurance measures. Data cleaning and analysis was done by Ferdous Hakim (FH) and MM with the guidance of MMZ. MMZ critically interpreted results. SAS conceptualized prepared the first draft of the manuscript, which was critically reviewed, revised and finalized by MMZ, MM, MRB, and FH. AHMEH is the guarantor of data. MMZ has guided the whole study.

Competing Interests: The authors of this study declare no conflict of interest. The authors alone are responsible for the views expressed in this article, and they do not necessarily represent the views, decisions or policies of the institutions with which they are affiliated.

Funding: The WHO Country Office for Bangladesh provided financial assistance for this study (WHO Reference: 2013/355662-0, Purchase Order: 200843353, Reg. File: BAN-2013-B7-TSA-0001). However, no fund has been used for preparing this manuscript.

Consent to publish: All authors consent to the publication of this manuscript.

Availability of data and materials: Data are available on reasonable request. Please contact Professor M. Mostafa Zaman at zamanm@who.int.

For peer review only

Table 1: Socio-demographic characteristics and relevant risk factors of the respondents, n (%)

Variables	Both (n=6391)	Men (n=2955)	Women (n=3436)
Age group (years)*			
<55	3684 (57.6)	1642 (55.6)	2042 (59.4)
≥55	2707 (42.4)	1313 (44.4)	1394 (40.6)
Residence			
Urban	1922 (30.1)	841 (28.5)	1081 (31.5)
Rural	4469 (69.9)	2114 (71.5)	2355 (68.5)
Education			
No formal schooling	3238 (50.9)	1147 (38.9)	2091 (61.1)
Any primary (classes 1–5)	1733 (27.2)	862 (29.3)	871(25.5)
Above primary (classes ≥6)	1397 (21.9)	937 (31.8)	460 (13.4)
Occupation			
Professional employee[†]	1015 (15.9)	886 (30.1)	129 (3.8)
Industrial worker/ Day laborer	1587 (24.9)	1430 (48.6)	157 (4.6)
Homemaker	2716 (42.6)	0 (0.0)	2716 (79.2)
Unemployed/ Retired	901 (14.1)	503 (17.1)	398 (11.6)
Others[‡]	153 (2.4)	124 (4.2)	29 (0.8)
Tobacco use (smoking or smokeless)	4066 (63.6)	2122 (71.8)	1944 (56.6)
Alcohol use, last 30 days	77 (1.2)	69 (2.3)	8 (0.2)
Overweight/ obesity[§]	1300 (20.5)	455 (15.5)	845 (24.7)
Diabetes mellitus[ǁ]	498 (7.8)	230 (7.8)	268 (7.8)
Hypertension[¶]	1623 (25.4)	689 (23.3)	934 (27.2)

Missing data for education, 23; occupation, 19; current tobacco use, 15; alcohol use in last 30 days, 21; body mass index, 32; diabetes mellitus, 8.

[*] Cut-off based on mean age (54.3 years).

[†] Professional employment: government and private company employee, businessman.

[‡] Others: shop keeper, weaver, driver, beggar, cook, carpenter, and tailor.

[§] Body mass index ≥25Kg/m^2; 1 pregnant woman was excluded.

[ǁ] Diabetes mellitus: random capillary blood glucose ≥11.1mmol/L and/ or known history of diabetes; 1 pregnant woman was excluded.

¶ Hypertension: blood pressure ≥140/90 mgHg or on medication for hypertension.

For peer review only - http://bmjopen.bmj.com/site/about/guidelines.xhtml

Table 2: Prevalence (%) of corrected visual acuities, percent (95% confidence interval)

Characteristics	Number (n=6391)	Normal (≥6/12) (n=5628)	Low vision (≥3/60-<6/60) (n=707)	Blind (<3/60) (n=56)
Age group, years				
<55	3684	98.1 (97.6–98.6)	1.7 (1.2–2,2)	0.2 (0.01–0.4)
≥55	2707	74.4 (71.4–77.4)	23.8 (20.9–26.7)	1.8 (1.1–2.5)
Sex				
Men	2955	87.2 (85.1–89.3)	12.0 (10.0–14.1)	0.7 (0.4–1.1)
Women	3436	88.8 (87.4–90.2)	10.2 (8.9–11.6)	1.0 (0.6–1.4)
Residence				
Urban	1922	87.7 (85.2–90.3)	11.8 (9.2–14.3)	0.5 (0.2–0.9)
Rural	4469	88.2 (86.3–90.1)	10.8 (8.9–12.6)	1.0 (0.6–1.4)
**Overall**	**6391**	**88.1 (86.5–89.5)**	**11.1 (9.6–12.6)**	**0.9 (0.6–1.2)**
**Overall (age adjusted) ***		**86.9 (85.2–88.6)**	**12.1 (10.5–13.8)**	**1.0 (0.5–1.4)**

* Adjusted for WHO World Population 2000-2020.[14]

For peer review only - http://bmjopen.bmj.com/site/about/guidelines.xhtml

For peer review only

Table 3: Odds ratios of risk factors for impaired vision and blindness after correction in Bangladeshi adults (n=6391)

Factors		Vision categories		Odds ratio (95% confidence interval)	
		Low vision and blind (<6/12) (n=763)	Normal vision (≥6/12) (n=5628)	Unadjusted	Adjusted for age and sex
Age, years	≥55	693 (90.8)	2014 (35.8)	17.8 (13.8–22.9)*	-
(≥55=1, <55=0)	<55	70 (9.2)	3614 (64.2)	1.0	-
Sex	Men	378 (49.5)	2577 (45.8)	1.2 (1.0–1.4)*	-
(man=1, woman=0)	Women	385 (50.5)	3051 (54.2)	1.0	-
Diabetes mellitus†	Yes	64 (8.4)	435 (7.7)	1.1 (0.8–1.4)	1.0 (0.7–1.3)
(yes=1, no=0)	No	698 (91.6)	5186 (92.3)	1.0	1.0
Hypertension	Yes	192 (25.2)	1431 (25.4)	1.0 (0.8–1.2)	0.8 (0.6–0.9)
(yes=1, no=0)	No	571 (74.8)	4197 (74.6)	1.0	1.0
Cataract	Yes	643 (84.3)	822 (14.6)	31.3 (25.4–38.6)*	17.0 (13.7–21.2)*
(yes=1, no=0)	No	120 (15.7)	4806 (85.4)	1.0	1.0
Diabetic retinopathy	Yes	31 (4.1)	80 (1.4)	2.9 (1.9–4.5)*	2.2 (1.4–3.5)*
(yes=1, no=0)	No	732 (95.9)	5548 (98.6)	1.0	1.0
Glaucoma	Yes	13 (1.7)	40 (0.7)	2.4 (1.3–4.5)*	1.4 (0.7–2.7)
(yes=1, no=0)	No	750 (98.3)	5588 (99.3)	1.0	1.0
AMD‡	Yes	12 (1.6)	17 (0.3)	5.3 (2.5–11.1)*	5.2 (2.1–12.7)*
(yes=1, no=0)	No	751 (98.4)	5611 (99.7)	1.0	1.0
Corneal disease	Yes	6 (0.8)	47 (0.8)	0.9 (0.4–2.2)	0.9 (0.4–2.4)
(yes=1, no=0)	No	757 (99.2)	5581 (99.2)	1.0	1.0
Ocular trauma	Yes	3 (0.4)	7 (0.1)	3.2 (0.8–12.3)	3.4 (0.7–16.6)
(yes=1, no=0)	No	760 (99.6)	5621 (99.9)	1.0	1.0
Eye lid disorder	Yes	4 (0.5)	21 (0.4)	1.4 (0.5–4.1)	0.6 (0.2–1.9)
(yes=1, no=0)	No	759 (99.5)	5607 (99.6)	1.0	1.0

† 8 missing values.

‡ AMD: age related macular degeneration.

* *P*<0.01

For peer review only

Figure 1: Flowchart for subject selection of the cross-sectional national survey done in urban and rural areas of all seven divisions in Bangladesh (n=6391)

.*HH indicates household; **PSU, primary sampling unit.

Figure 2. Prevalence of blindness according to age groups among the respondents of the cross-sectional national survey on visual impairments in Bangladesh (n=6391)

Figure 3. Prevalence of various eye conditions among the respondents of the cross-sectional national survey on visual impairments in Bangladesh (n=6391)

For peer review only

References

1. West S, Sommer A. Prevention of blindness and priorities for the future. *Bulletin of the World Health Organization*. 2001;79:244-248
2. Pascolini D, Mariotti SP. Global estimates of visual impairment: 2010. *The British journal of ophthalmology*. 2012;96:614-618
3. World health organization. Blindness and vision impairment. Fact sheet. 2011 [internet] [cited 2011 oct. 8] world health organization. Available from: Http://www.Who.Int/blindness/en/
4. Ministry of health and family welfare, national council for blind. National eye care plan for implementation of vision 2020 in bangladesh. [internet] ministry of health and family welfare, government of the people's republic of bangladesh. [cited 2020 may 27] available from: Http://nec.Gov.Bd/opr_pdf/national_eye_care_plan.Pdf
5. Dineen BP, Bourne RR, Ali SM, Huq DM, Johnson GJ. Prevalence and causes of blindness and visual impairment in bangladeshi adults: Results of the national blindness and low vision survey of bangladesh. *The British journal of ophthalmology*. 2003;87:820-828
6. Sight savers. Bangladesh quadruples its eye care budget. [accessed 28 october 28, 2020]. Https://www.Sightsavers.Org/news/2017/06/bangladesh-eye-care-budget/
7. Rosser DA, Laidlaw DA, Murdoch IE. The development of a "reduced logmar" visual acuity chart for use in routine clinical practice. *The British journal of ophthalmology*. 2001;85:432-436
8. Kish L. A procedure for objective respondent selection within the household. *Journal of the American Statistical Association*. 1949;44:380-387
9. Who programme for the prevention of blindness. (1988). Coding instructions for the who/pbl eye examination record (version iii).World health organization. [cited 2020 may 27] available from: Https://apps.Who.Int/iris/handle/10665/67896.
10. Fletcher AE, Ellwein LB, Selvaraj S, Vijaykumar V, Rahmathullah R, Thulasiraj RD. Measurements of vision function and quality of life in patients with cataracts in southern india. Report of instrument development. *Archives of ophthalmology (Chicago, Ill. : 1960)*. 1997;115:767-774
11. World health organization. Blindness and vision impairment: Definitions. [internet]. Available from: Https://www.Who.Int/news-room/fact-sheets/detail/blindness-and-visual-impairment [accessed: 17 june 2020].
12. Dunn G. *Design and analysis of reliability studies: The statistical evaluation of measurement errors*. London, England: Edward Arnold Publishers; 1989.

For peer review only - http://bmjopen.bmj.com/site/about/guidelines.xhtml

13. Foster PJ, Buhrmann R, Quigley HA, Johnson GJ. The definition and classification of glaucoma in prevalence surveys. *The British journal of ophthalmology*. 2002;86:238-242
14. Ahmad ob, boschi-pinto c, lopez ad, et al. Age standardization of rates: A new who standard. Gpe discussion paper series: No31 geneva, switzerland: World health organization, 2001. .
15. Gaur A. Demographic transition of bangladesh. *International Journal of Science and Research* 2019;8:666-670
16. Wong TY, Zheng Y, Wong W-L, III ELL, Wang J-J, Mitchell P, Cheung N, Aung T, Saw SM, Cheng CY. The prevalence and causes of visual impairment and blindness in a multi-ethnic asian population: The singapore epidemiology of eye disease (seed) study. *Investigative Ophthalmology & Visual Science*. 2012;53:5640-5640
17. Liu JH, Cheng CY, Chen SJ, Lee FL. Visual impairment in a taiwanese population: Prevalence, causes, and socioeconomic factors. *Ophthalmic epidemiology*. 2001;8:339-350
18. Zainal M, Ismail SM, Ropilah AR, Elias H, Arumugam G, Alias D, Fathilah J, Lim TO, Ding LM, Goh PP. Prevalence of blindness and low vision in malaysian population: Results from the national eye survey 1996. *The British journal of ophthalmology*. 2002;86:951-956
19. Xu L, Wang Y, Li Y, Wang Y, Cui T, Li J, Jonas JB. Causes of blindness and visual impairment in urban and rural areas in beijing: The beijing eye study. *Ophthalmology*. 2006;113:1134.e1131-1111
20. Bourne RRA, Flaxman SR, Braithwaite T, Cicinelli MV, Das A, Jonas JB, Keeffe J, Kempen JH, Leasher J, Limburg H, et al. Magnitude, temporal trends, and projections of the global prevalence of blindness and distance and near vision impairment: A systematic review and meta-analysis. *The Lancet. Global health*. 2017;5:e888-e897
21. Jadoon MZ, Dineen B, Bourne RR, Shah SP, Khan MA, Johnson GJ, Gilbert CE, Khan MD. Prevalence of blindness and visual impairment in pakistan: The pakistan national blindness and visual impairment survey. *Invest Ophthalmol Vis Sci*. 2006;47:4749-4755
22. Baasanhu J, Johnson GJ, Burendei G, Minassian DC. Prevalence and causes of blindness and visual impairment in mongolia: A survey of populations aged 40 years and older. *Bulletin of the World Health Organization*. 1994;72:771-776
23. Saw SM, Husain R, Gazzard GM, Koh D, Widjaja D, Tan DT. Causes of low vision and blindness in rural indonesia. *The British journal of ophthalmology*. 2003;87:1075-1078
24. Murthy GV, Gupta SK, Bachani D, Jose R, John N. Current estimates of blindness in india. *The British journal of ophthalmology*. 2005;89:257-260
25. Thapa R, Bajimaya S, Paudyal G, Khanal S, Tan S, Thapa SS, van Rens G. Prevalence and causes of low vision and blindness in an elderly population in nepal: The bhaktapur retina study. *BMC ophthalmology*. 2018;18:42

For peer review only - http://bmjopen.bmj.com/site/about/guidelines.xhtml

26. International centre for eye health, institute of ophthalmology london, national programme for the prevention of blindness federal ministry of health nigeria, national eye centre nigeria, sightsavers international nigeria. The nigeria national blindness and visual impairment survey 2005-2007. Available from: Blogs.Lshtm.Ac.Uk/iceh/files/2014/04/nigeriasurvey.Pdf.
27. Soori H, Ali JM, Nasrin R. Prevalence and causes of low vision and blindness in tehran province, iran. *JPMA. The Journal of the Pakistan Medical Association*. 2011;61:544-549
28. Limburg H, Barria von-Bischhoffshausen F, Gomez P, Silva JC, Foster A. Review of recent surveys on blindness and visual impairment in latin america. *The British journal of ophthalmology*. 2008;92:315-319
29. National institute of health. National eye institute. Age-related macular degeneration. [accessed 31 october 2020]. Https://www.Nei.Nih.Gov/learn-about-eye-health/eye-conditions-and-diseases/age-related-macular-degeneration
30. Chen MS, Kao CS, Chang CJ, Wu TJ, Fu CC, Chen CJ, Tai TY. Prevalence and risk factors of diabetic retinopathy among noninsulin-dependent diabetic subjects. *American journal of ophthalmology*. 1992;114:723-730
31. Dandona L, Dandona R, Naduvilath TJ, McCarty CA, Rao GN. Population based assessment of diabetic retinopathy in an urban population in southern india. *The British journal of ophthalmology*. 1999;83:937-940
32. Nirmalan PK, Katz J, Robin AL, Tielsch JM, Namperumalsamy P, Kim R, Narendran V, Ramakrishnan R, Krishnadas R, Thulasiraj RD, et al. Prevalence of vitreoretinal disorders in a rural population of southern india: The aravind comprehensive eye study. *Archives of ophthalmology (Chicago, Ill. : 1960)*. 2004;122:581-586
33. Wong TY, Loon SC, Saw SM. The epidemiology of age related eye diseases in asia. *The British journal of ophthalmology*. 2006;90:506-511
34. Vijaya L, George R, Asokan R, Velumuri L, Ramesh SV. Prevalence and causes of low vision and blindness in an urban population: The chennai glaucoma study. *Indian journal of ophthalmology*. 2014;62:477-481
35. Asbell PA, Dualan I, Mindel J, Brocks D, Ahmad M, Epstein S. Age-related cataract. *The Lancet*. 2005;365:599-609
36. Prokofyeva E, Wegener A, Zrenner E. Cataract prevalence and prevention in europe: A literature review. *Acta ophthalmologica*. 2013;91:395-405

For peer review only - http://bmjopen.bmj.com/site/about/guidelines.xhtml

Figure 1

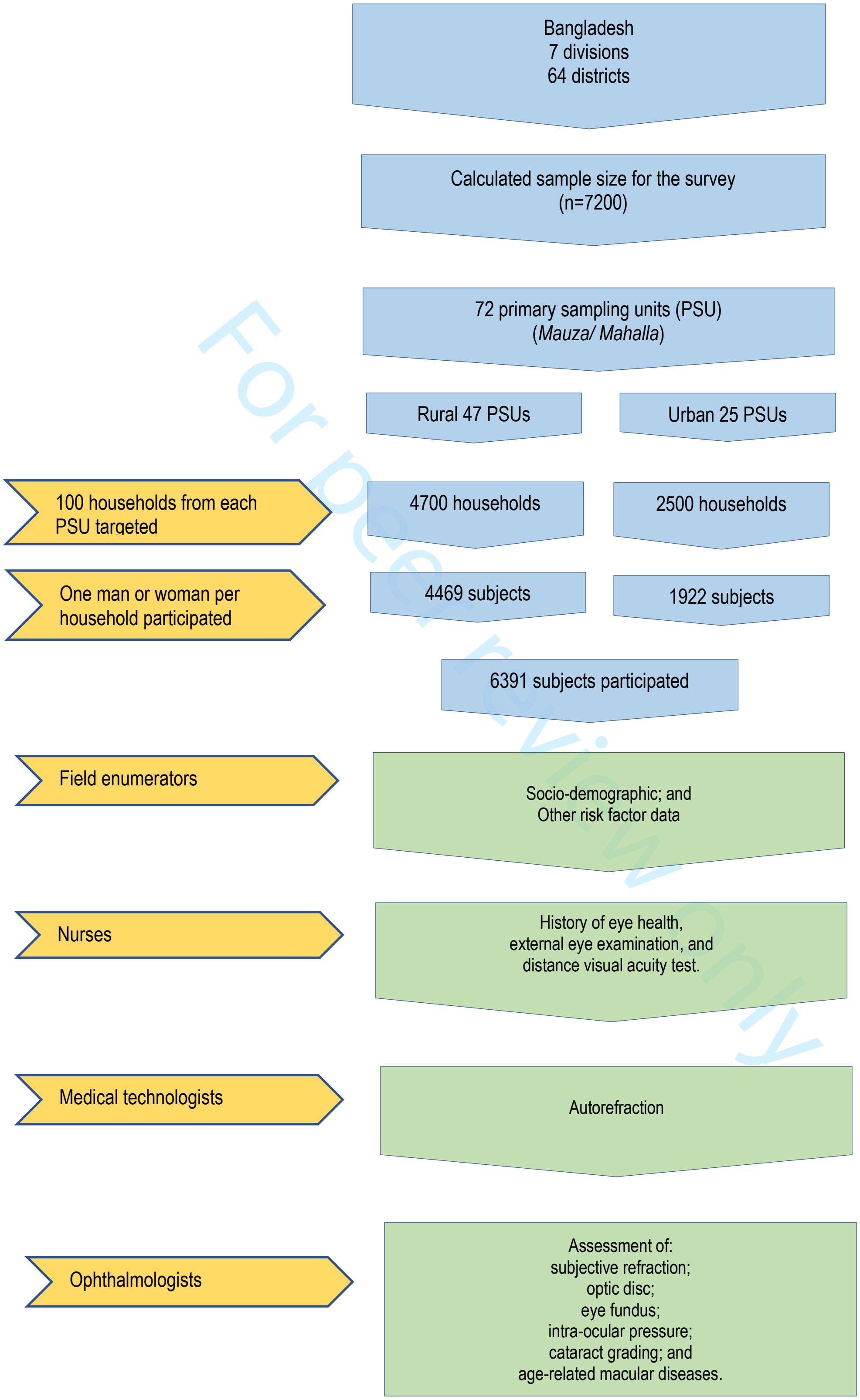
Bangladesh
7 divisions
64 districts
Calculated sample size for the survey
(n=7200)
72 primary sampling units (PSU)
(Mauza/ Mahalla)
Rural 47 PSUs
Urban 25 PSUs
100 households from each PSU targeted
4700 households
2500 households
One man or woman per household participated
4469 subjects
1922 subjects
6391 subjects participated
Field enumerators
Socio-demographic; and
Other risk factor data
Nurses
History of eye health,
external eye examination, and
distance visual acuity test.
Medical technologists
Autorefraction
Ophthalmologists
Assessment of:
subjective refraction;
optic disc;
eye fundus;
intra-ocular pressure;
cataract grading; and
age-related macular diseases.

Figure 2

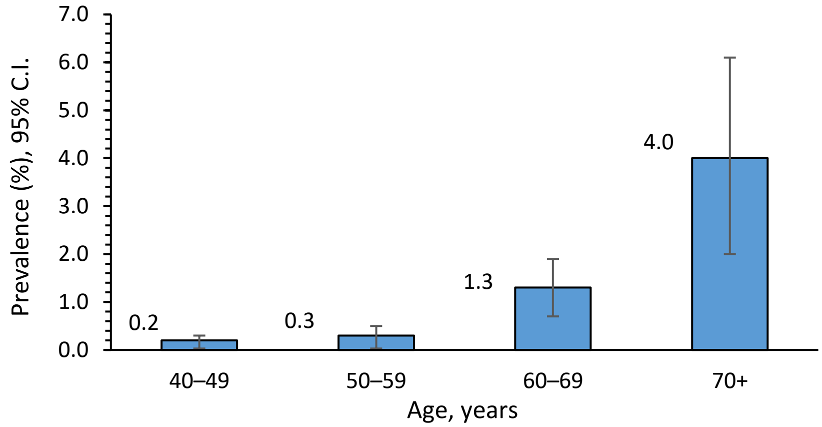

For peer review only

Figure 3

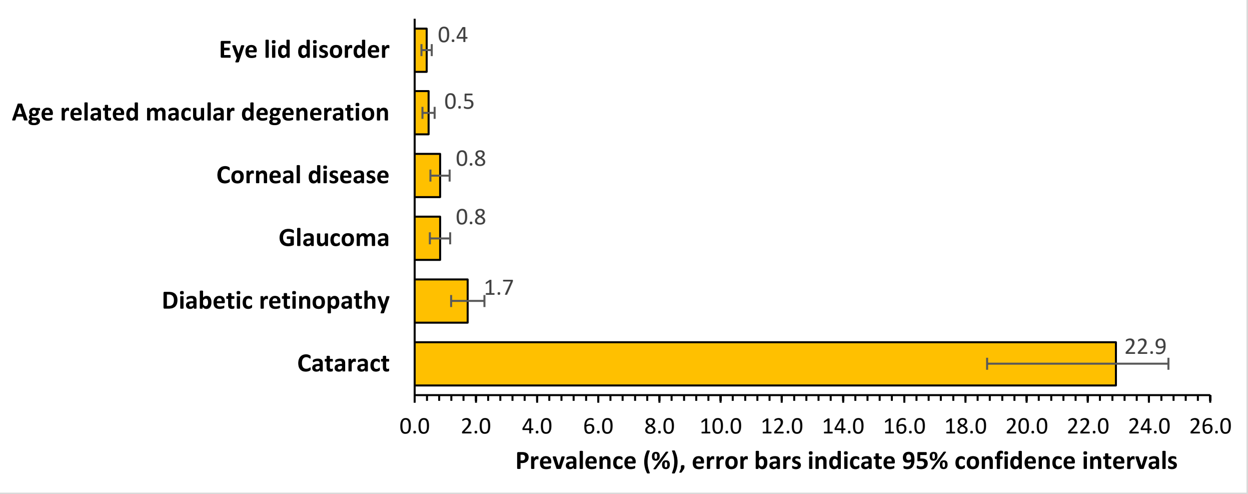

peer review only

STROBE (Strengthening The Reporting of OBservational Studies in Epidemiology) Checklist

A checklist of items that should be included in reports of observational studies. You must report the page number in your manuscript where you consider each of the items listed in this checklist. If you have not included this information, either revise your manuscript accordingly before submitting or note N/A.

Note: An Explanation and Elaboration article discusses each checklist item and gives methodological background and published examples of transparent reporting. The STROBE checklist is best used in conjunction with this article (freely available on the Web sites of PLoS Medicine at http://www.plosmedicine.org/, Annals of Internal Medicine at http://www.annals.org/, and Epidemiology at http://www.epidem.com/). Information on the STROBE Initiative is available at www.strobe-statement.org.

Section and Item	Item No.	Recommendation	Reported on Page No.
**Title and Abstract**	1	(*a*) Indicate the study's design with a commonly used term in the title or the abstract	
		(*b*) Provide in the abstract an informative and balanced summary of what was done and what was found	
**Introduction**			
Background/Rationale	2	Explain the scientific background and rationale for the investigation being reported	
Objectives	3	State specific objectives, including any prespecified hypotheses	
**Methods**			
Study Design	4	Present key elements of study design early in the paper	
Setting	5	Describe the setting, locations, and relevant dates, including periods of recruitment, exposure, follow-up, and data collection	
Participants	6	(*a*) *Cohort study*—Give the eligibility criteria, and the sources and methods of selection of participants. Describe methods of follow-up<br>*Case-control study*—Give the eligibility criteria, and the sources and methods of case ascertainment and control selection. Give the rationale for the choice of cases and controls<br>*Cross-sectional study*—Give the eligibility criteria, and the sources and methods of selection of participants	
		(*b*) *Cohort study*—For matched studies, give matching criteria and number of exposed and unexposed<br>*Case-control study*—For matched studies, give matching criteria and the number of controls per case	
Variables	7	Clearly define all outcomes, exposures, predictors, potential confounders, and effect modifiers. Give diagnostic criteria, if applicable	

For peer review only

Section and Item	Item No.	Recommendation	Reported on Page No.
Data Sources/ Measurement	8*	For each variable of interest, give sources of data and details of methods of assessment (measurement). Describe comparability of assessment methods if there is more than one group	
Bias	9	Describe any efforts to address potential sources of bias	
Study Size	10	Explain how the study size was arrived at	
Quantitative Variables	11	Explain how quantitative variables were handled in the analyses. If applicable, describe which groupings were chosen and why	
Statistical Methods	12	(*a*) Describe all statistical methods, including those used to control for confounding	
		(*b*) Describe any methods used to examine subgroups and interactions	
		(*c*) Explain how missing data were addressed	
		(*d*) *Cohort study*—If applicable, explain how loss to follow-up was addressed<br>*Case-control study*—If applicable, explain how matching of cases and controls was addressed<br>*Cross-sectional study*—If applicable, describe analytical methods taking account of sampling strategy	
		(*e*) Describe any sensitivity analyses	
**Results**			
Participants	13*	(a) Report numbers of individuals at each stage of study—eg numbers potentially eligible, examined for eligibility, confirmed eligible, included in the study, completing follow-up, and analysed	
		(b) Give reasons for non-participation at each stage	
		(c) Consider use of a flow diagram	
Descriptive Data	14*	(a) Give characteristics of study participants (eg demographic, clinical, social) and information on exposures and potential confounders	
		(b) Indicate number of participants with missing data for each variable of interest	
		(c) *Cohort study*—Summarise follow-up time (eg, average and total amount)	
Outcome Data	15*	*Cohort study*—Report numbers of outcome events or summary measures over time	
		Case-control study—Report numbers in each exposure category, or summary measures of exposure	
		Cross-sectional study—Report numbers of outcome events or summary measures	

For peer review only

Section and Item	Item No.	Recommendation	Reported on Page No.
Main Results	16	(*a*) Give unadjusted estimates and, if applicable, confounder-adjusted estimates and their precision (eg, 95% confidence interval). Make clear which confounders were adjusted for and why they were included	
		(*b*) Report category boundaries when continuous variables were categorized	
		(*c*) If relevant, consider translating estimates of relative risk into absolute risk for a meaningful time period	
Other Analyses	17	Report other analyses done—eg analyses of subgroups and interactions, and sensitivity analyses	
**Discussion**			
Key Results	18	Summarise key results with reference to study objectives	
Limitations	19	Discuss limitations of the study, taking into account sources of potential bias or imprecision. Discuss both direction and magnitude of any potential bias	
Interpretation	20	Give a cautious overall interpretation of results considering objectives, limitations, multiplicity of analyses, results from similar studies, and other relevant evidence	
Generalisability	21	Discuss the generalisability (external validity) of the study results	
**Other Information**			
Funding	22	Give the source of funding and the role of the funders for the present study and, if applicable, for the original study on which the present article is based	

*Give information separately for cases and controls in case-control studies and, if applicable, for exposed and unexposed groups in cohort and cross-sectional studies.

Once you have completed this checklist, please save a copy and upload it as part of your submission. DO NOT include this checklist as part of the main manuscript document. It must be uploaded as a separate file.

For peer review only
